# Ability to identify patient-ventilator asynchronies in intensive care unit professionals: A multicenter cross-sectional analytical study

**DOI:** 10.2478/jccm-2025-0017

**Published:** 2025-04-30

**Authors:** Andrés Mauricio Enríquez Popayán, Iván Ignacio Ramírez, Juan Felipe Zúñiga, Ruvistay Gutierrez-Arias, Mayda Alejandra Jiménez Pérez, Henry Mauricio Parada-Gereda, Luis Fernando Pardo Cocuy, Ana Lucia Rangel Colmenares, Nubia Castro Chaparro, Ana Pinza Ortega, Jorge Martínez Díaz, Johanna Hurtado Laverde, Joismer Alejandro Henao Cruz

**Affiliations:** Departamento de Fisioterapia en Cuidado Intensivo, GyO Medical, Yopal, Colombia; Departamento de Fisioterapia en Cuidado Intensivo, Clínica SIMALINK, Yopal, Colombia; Departamento de Apoyo en Rehabilitación Cardiopulmonar Integral, Instituto Nacional del Tórax, Santiago, Chile; INTRehab Research Group, Instituto Nacional del Tórax, Santiago, Chile; Escuela de Kinesiología, Facultad de Ciencias de la Salud, Universidad Diego Portales, Santiago, Chile; Departamento de Fisioterapia en Cuidado Intensivo, Clínica de Occidente, Cali, Colombia; Departamento de Apoyo en Rehabilitación Cardiopulmonar Integral, Instituto Nacional del Tórax, Santiago, Chile; INTRehab Research Group, Instituto Nacional del Tórax, Santiago, Chile; Exercise and Rehabilitation Sciences Institute, Faculty of Rehabilitation Sciences, Universidad Andres Bello, Santiago, Chile; Departamento de Fisioterapia en Cuidado Intensivo, Fundación Hospital San Pedro, Pasto, Colombia; Unidad de Cuidado Intensivo, Clínica Reina Sofia, Bogotá, Colombia; Clínica Colsanitas, Grupo de Investigación en Nutrición Clínica y Rehabilitación-Keralty, Bogotá, Colombia. Especialización de Fisioterapia en Paciente Adulto Critico, Universidad del Rosario, Bogotá, Colombia; Departamento de Fisioterapia en Cuidado Intensivo, Hospital Universitario Mayor Méderi, Bogotá, Colombia; Especialización de Fisioterapia en Paciente Adulto Critico, Universidad del Rosario, Bogotá, Colombia; Departamento de Fisioterapia en Cuidado Intensivo, Clínica Norte, Cúcuta, Colombia; Departamento de Fisioterapia en Cuidado Intensivo, Clínica San José, Cúcuta, Colombia; Departamento de Cuidado Intensivo, Lumira SAS, Cali, Colombia; Departamento de Fisioterapia en Cuidado Intensivo, Fundación Hospital San Pedro, Pasto, Colombia; Programa de Fisioterapia, Universidad Mariana, Pasto, Colombia; Grupo de Investigación en Medicina Critica GMC, Fundación Hospital San Pedro, Pasto, Colombia; Departamento de Cuidado Intensivo, Fundación Hospital San Pedro, Pasto, Colombia; Programa de Medicina, Fundación Universidad San Martin, Pasto, Colombia; Grupo de Investigación en Medicina Critica GMC, Fundación Hospital San Pedro, Pasto, Colombia; Programa de Terapia Respiratoria, Universidad Manuela Beltrán, Bogotá, Colombia; Departamento de Fisioterapia en Cuidado Intensivo, Hospital Universitario de Caldas, Manizales, Colombia

**Keywords:** mechanical ventilation, critical care, patient-ventilator interaction, patient-ventilator asynchrony

## Abstract

**Introduction:**

Patient-ventilator asynchrony (PVA) is frequent in intensive care. Its presence is associated with prolonged days of mechanical ventilation and may lead to increased mortality in the intensive care unit (ICU) and hospital. Little is known about the ability of Colombian intensive care professionals to identify asynchronies, and the factors associated with their correct identification are not apparent.

**Aim of the study:**

To describe the ability of Colombian intensive care professionals to identify patient-ventilator asynchronies (PVA) using waveform analysis. In addition, to define the characteristics associated with correctly detecting PVA.

**Material and methods:**

We conducted a multicenter, cross-sectional, national survey-based study between January and August 2024. Colombian physiotherapists, respiratory therapists, nurses and intensive care physicians from 24 departments participated in the study. An online survey was used. They were asked to identify six different PVAs presented as videos. The videos were displayed using pressure/time and flow/time waveform of a Puritan Bennett 840 ventilator.

**Results:**

We recruited 900 participants, 60% female, most of whom were physiotherapists (53%). Most professionals had specialty training in critical care (42%), and 32% reported having specific PVA training. Double triggering was the most frequently identified PVA (75%). However, only 3.67% of participants recognized all six PVAs. According to multiple logistic regression analysis, working in a mixed unit (OR 2.59; 95% CI 1.19 – 5.54), caring for neonates (OR 5.19; 95% CI 1.77 – 15.20), and having specific training (OR 2.38; 95% CI 1.16 – 4.76) increases the chance of correctly recognizing all PVAs.

**Conclusion:**

In Colombia, a low percentage of professionals recognize all PVAs. Having specific training in this topic, working in mixed ICUs and neonatal intensive care was significantly associated with identifying all PVAs.

## Introduction

Patient-ventilator asynchrony (PVA) is common in intensive care [1,2]. Between 25% and 93% of patients may experience at least one episode of PVA [3,4]. The incidence of PVA has been associated with prolonged durations of mechanical ventilation (MV) and may contribute to increased mortality in both the intensive care unit (ICU) and the hospital [5]. This effect may depend on the intensity and duration of exposure to PVA during MV [6].

Various methods are available to identify PVA, with waveform analysis of MV being the most extensively studied and commonly used in clinical practice [7]. Studies assessing this skill indicate that proficiency among intensive care professionals is generally below 30% [8,9]. Even with specific training programmes, competency rates do not exceed 70% [10].

Few studies have explored the factors associated with accurate PVA recognition. However, specialised training in this area, including courses exceeding 100 hours, has shown a significant positive correlation [11]. Studies assessing this competency often face limitations, such as small sample sizes and underrepresenting ICU professionals from specific regions or countries.

We conducted the first national survey to describe the ability of Colombian ICU professionals to identify PVA through waveform analysis and examine the characteristics associated with accurate PVA detection.

## Methods

This study complies with international guidelines, including the Declaration of Helsinki, the Nuremberg Code, and Colombian research standards regarding informed consent, data protection, and risk classification (Resolution 8430 of 1993 from the Ministry of Health). It was approved by the Health Research Ethics Committee (CEIS) of the Hospital Regional de la Orinoquía (Act 037, 29 September 2023).

### Study design and participants

A multicenter cross-sectional study was conducted from January to August 2024. The questionnaire, administered via an online survey (Google Forms), required participants to identify six types of PVA. Physiotherapists, respiratory therapists, nurses, and physicians working in Colombian ICUs were invited to participate. Incomplete survey responses were excluded from the analysis.

### Survey

The survey was structured into three sections. The first section outlined the study’s objective and gathered sociodemographic and professional information about each participant, including age, gender, profession, additional training, work experience, and service experience. The second section focused on professional habits, such as the frequency of waveform monitoring, while the third section assessed participants’ ability to identify PVA in six video recordings.

**Fig. 1.**
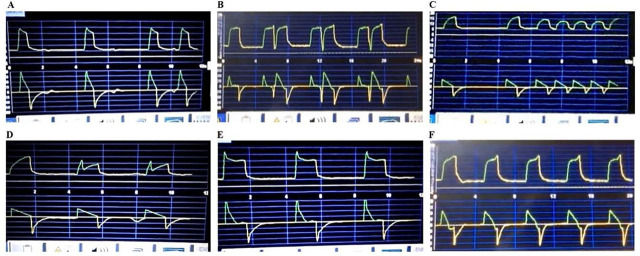
**Ventilatory asynchronies evaluated.** A: Ineffective triggering; B: Double triggering; C: Auto–triggering; D: Insufficient flow; E: Excessive flow; F: Delayed cycling

The instrument featured six PVA videos displaying pressure/time and flow/time waveforms (Figure 1). Ten MV experts validated each video, achieving 100% inter-observer agreement. All PVA recordings were captured from a Puritan Bennett 840 mechanical ventilator (Covidien, Carlsbad, CA, USA).

### Statistical analysis

Categorical variables were summarised using absolute and relative frequencies, and quantitative variables were described by medians and interquartile ranges.

Simple and multiple logistic regression analyses were conducted to identify factors associated with the likelihood of correctly identifying all PVAs. In both models, the response variable was the identification of PVAs, categorised as 0: identifying five or fewer PVAs and 1: identifying all six PVAs. The backward elimination method was used for multiple regression, removing variables with a p-value greater than 0.1. The initial model included sex, age, years of experience, profession, type of ICU, target population, type of postgraduate training, and specific training in PVA. Odds ratios (OR) and their 95% confidence intervals (95% CI) were reported.

The analyses were performed using JASP software (JASP Team, 2024, Version 0.19.1), with a 5% statistical significance threshold.

## Results

**Fig. 2.**
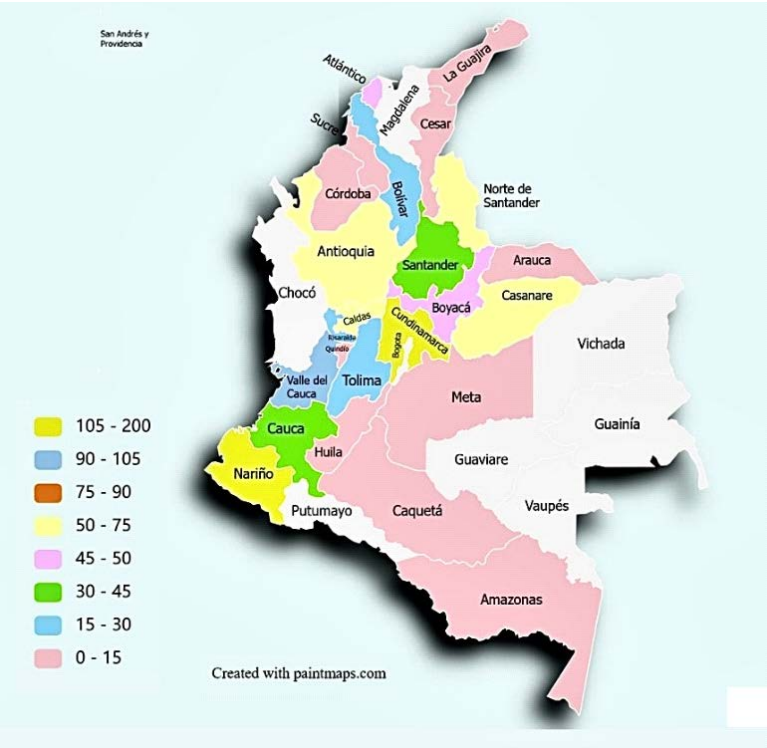
Distribution of study participants by department in Colombia.

The study included 900 Colombian ICU professionals, of whom 59.56% were women. The median age was 32 years (IQR 28–38). The majority were physiotherapists (53.11%), followed by respiratory therapists (22.11%), physicians (20.56%), and nurses (4.22%) ( Table 1). Participants represented 24 departments, covering 75% of Colombia’s regions, with the highest representation from Cundinamarca (15%) and Nariño (14%) (Figure 2).

**Table 1.** Characteristics of study participants.

**Variable**	**Result (n = 900)**
Female, n (%)	536 (59.56)
Age (years), median (IQR)	32 (28 – 38)

**Profession, n (%)**	
Nursing	38 (4.22)
Physiotherapy	478 (53.11)
Medicine	185 (20.56)
Respiratory therapy	199 (22.11)
Private institution, n (%)	531 (59)

**Postgraduate training, n (%)**	
Course	80 (8.89)
Graduate	299 (33.22)
PhD	1 (0.11)
Training	60 (6.67)
Specialty	381 (42.33)
Master	50 (5.56)
None	29 (3.22)
Clinical postgraduate training, n (%)	850 (94.44)
Work experience (year), median (IQR)	6 (3 – 11)
Mixed ICU, n (%)	426 (47.33)
Specific PVA training, n (%)	286 (31.78)

**Population served, n (%)**	
Adults	811 (90.11)
Neonates	49 (5.44)
Paediatrics	40 (4.44)
Number of beds, median (IQR)	13 (10 – 20)
Patients in MV per day, median (IQR)	6 (4 – 9)

***MV adjustment frequency, n (%)***	
1–2 times during the workday	564 (62.67)
3–4 times during the workday	185 (20.56)
5–6 times during the workday	39 (4.33)
7–8 times during the workday	11 (1.22)
9–10 times during the workday	5 (0.56)
More than 10 times during the workday	8 (0.89)
I do not make adjustments	80 (8.89)
I do not feel confident making adjustments	8 (0.89)

**Frequency of waveform monitoring in MV, n (%)**	
1 time during a 6-hour shift	184 (20.44)
1 time during a 12-hour shift	26 (2.89)
2 times during a 6-hour shift	85 (9.44)
2 times during a 12-hour shift	72 (8)
3 times during a 12-hour shift	68 (7.56)
Every 1 hour	97 (10.78)
Every 2 hours	169 (18.78)
Every 3 hours	101 (11.22)
Do not waveform analysis MV	98 (10.89)

ICU: Intensive care unit, IQR: Interquartile range, MV: Mechanical ventilation, PVA: Patient-ventilator asynchronies, n: number of responses, %: percentage, PhD: Doctorate

A total of 42.33% of participants reported completing a specialised programme in intensive care, and 32% had received specific training in PVA. The median work experience was six years (IQR 3–11). Most participants worked in mixed (47.33%) and adult ICUs (90.11%). Half of the ICUs where participants were employed had 13 or more beds (IQR 10–20), with MV patients occupying six or more beds (IQR 4–9). Most professionals reported monitoring patient waveforms once during a six-hour working shift (20.44%) and making one to two adjustments to MV settings (62.67%).

**Fig. 3.**
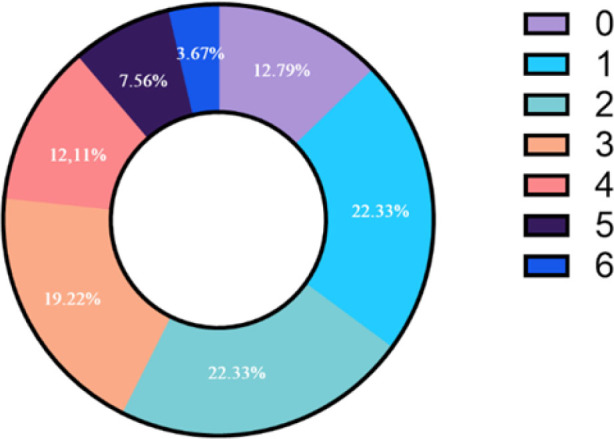
Proportion of participants by number of correctly identified PVA.

The professionals most frequently identified double triggering (75%), while late cycling was identified less often (22.89%) (Table 2). Additionally, 12.79% of participants failed to identify any PVA, whereas 3.67% were able to identify all PVAs (Figure 3). Simple logistic regression analysis revealed that working in a mixed ICU (OR 2.59; 95% CI 1.10–4.79), caring for neonates (OR 3.18; 95% CI 1.17–8.63), and receiving specific training in PVA identification (OR 2.38; 95% CI 1.18–4.76) were factors that increased the likelihood of correctly identifying all PVAs (Table 3). The multiple regression analysis further confirmed that these three characteristics significantly increased the chances of correctly identifying all PVAs (Table 4).

**Table 2.** Proportion of professionals who correctly identified each of the PVAs.

**PVA**	**Proper identification, n (%)**
Double triggering	675 (75)
Late cycling	206 (22,89)
Auto-triggering	380 (42,22)
Insufficient flow	296 (32,89)
Ineffective triggering	261 (29)
Excessive flow	278 (30,89)

**PVA:** Patient-ventilator asynchronies, **n:** number of responses, **%**: percentage

**Table 3.** Factors associated with proper identification of all PVAs (Simple logistic regression).

**Variable**	**OR (CI 95%)**
Profession	
Physiotherapy	1
Medicine	0.44 (0.15 – 1.28)
Respiratory therapy	0.51 (0.19 – 1.36)
Nursing	0.54 (0.07 – 4.07)
Work experience (year)	0.99 (0.94 – 1.05)
Mixed ICU	2.59 (1.10 – 4.79)*

**Population served**	
Adult	1
Paediatrics	0 (0 - ∞)
Neonates	3.18 (1.17 – 8.63)*

**Frequency of waveform monitoring in MV, n (%)**	
1 time during a 6-hour shift	1
1 time during a 12-hour shift	0 (0 - ∞)
2 times during a 6-hour shift	0.53 (0.11 – 2.55)
2 times during a 12-hour shift	0.63 (0.13 – 3.03)
3 times during a 12-hour shift	0.33 (0.04 – 2.68)
Every 1 hour	0.95 (0.28 – 3.23)
Every 2 hours	1.38 (0.53 – 3.59)
Every 3 hours	1.39 (0.47 – 4.12)
Do not waveform analysis VM	0 (0 - ∞)
Number of beds	1.03 (0.99 – 1.06)
Patients in MV per day	1.02 (0.96 – 1.09)

**Postgraduate training, n (%)**	
Specialty	1
Course	0.46 (0.11 – 2.02)
Training	0 (0 - ∞)
Graduate	0.50 (0.22 – 1.14)
Master	1.15 (0.33 – 4.03)
PhD	0 (0 - ∞)
Specific PVA training, n (%)	2.38 (1.18 – 4.76)*

**PVA:** Patient-ventilator asynchronies. **ICU:** Intensive care unit, **MV:** Mechanical ventilation, **OR**: Odds ratio, **n:** number of responses, **%**: percentage, PhD: Doctorate, **∞**: Infinite

**Table 4.** Factors associated with proper identification of all PVAs (multiple logistic regression).

**Variable**	**OR (CI 95%)**
Mixed ICU	2.59 (1.19 – 5.54)
Population served (Neonates)	5.19 (1.77 – 15.20)
Specific PVA training, n (%)	2.38 (1.16 – 4.76)

**ICU:** Intensive care unit, **PVA:** Patient-ventilator asynchronies, **n:** number of responses, **%**: percentage, **OR**: Odds ratio

## Discussion

This study represents the first national survey assessing the ability of ICU professionals in Colombia to identify PVAs through mechanical ventilation waveform analysis. Professionals from 75% of Colombia’s departments participated.

In our study, 3.67% of participants correctly identified all six PVAs, a result that falls below the mean. The international survey by Ramirez et al. [11] found that 19.5% of participants identified the same six PVAs. National research reports provide similar data; the study by Alqahtani et al. [9] reported that 10.2% of participants correctly identified three of the PVAs evaluated in Saudi Arabia. In addition, the research carried out by Zelalem et al. [12] describes that in Ethiopia, only 10.5% of the professionals were able to identify the PVAs studied correctly.

The discrepancy in results may be linked to the inclusion of more remote regions in Colombia, where healthcare access is more limited, and the levels of care tend to be of lower complexity. Additionally, the sample size could be a contributing factor. In studies with smaller populations, participants with a particular interest in the topic are more likely to respond. In contrast, more extensive studies reflect better the broader reality, encompassing all professionals.

However, this ability also varies among staff working in intensive care. Studies that have evaluated this skill in specific professions provide data that draw attention. In physicians, this ability is only 52.9% [13], in nurses, 12% [14], and in respiratory therapists, 1.7% [15]. This information tells us that the national data is likely low. This is because the capacity to identify asynchronies is not integrated between each profession; therefore, the global data does not exceed 30%. This is also worrying because failure to identify asynchronies correctly could trigger complications associated with their presence, such as prolongation of mechanical ventilation [5].

Current studies, despite their methodological rigour, lack a broader population base. The article by Ramirez et al. includes 366 participants [8], Zelalem et al. has 237 [12], and Mohamed’s survey has 101 responses [14], meaning their results reflect a percentage of intensive care professionals. In contrast, our study collected 900 responses distributed across the entire national territory. This larger population allows for a more comprehensive assessment of the status of ICU professionals’ ability to identify PVAs.

The most frequently identified PVA was double triggering. Benítez et al. [16] noted that this PVA is among the most common and potentially fatal, making it highly prevalent in mechanically ventilated patients. Excess flow, on the other hand, was the PVA with the fewest correct identifications. Saavedra et al. [17] highlighted that this is a rare PVA with a low incidence, and many professionals are unfamiliar with its occurrence under ventilatory conditions. Notably, 12.79% of respondents failed to identify any PVA.

In this study, we identified new factors associated with correctly identifying PVAs, such as working in a mixed and neonatal ICU, where the latter population requires rigorous preparation from healthcare personnel. Hermanspann et al. [18] noted that neonatal ICU nurses make fewer errors than adult ICU nurses, and their high level of preparation likely enhances their capacity to identify PVAs. Specific training in this area was also significantly correlated with improved identification, aligning with previous findings of Ramírez et al. [8].

We found that MV waveforms are typically monitored once during six hours, with some professionals indicating they do not actively observe the monitor. The analysis of MV waveforms is crucial for accurate PVA detection, as the lack of regular monitoring in critically ill patients can have negative clinical consequences, including increased mortality and ICU admissions [19,20].

In daily practice, maintaining continuous 24-hour monitoring is challenging due to the other tasks that must be performed in the ICU. Therefore, software like IntelliSync+ is a valuable tool in complementing the care of critically ill patients. Nakornnoi et al. [21] describe how this program reduces inspiratory activation delay time compared to conventional systems, which can improve patient-ventilator synchrony.

Our results revealed that profession and years of experience in the ICU are not associated with correctly identifying PVAs, nor are the number of times the MV is adjusted or postgraduate training correlated with this skill.

The strengths of this study lie in the large number of professionals who responded to the survey, representing a diverse sample from various regions of the country. This broad participation allows for more generalisable results and provides a comprehensive and robust view of ICU professionals’ ability to identify PVAs. On the other hand, most of the participants in our study were physiotherapists. In some contexts, these professionals are only dedicated to rehabilitating physical function. However, in Colombia, as in many other Latin American countries, it is common for physiotherapists to have undergraduate training in respiratory care and to continue specialising in respiratory critical care and management of MV. This is probably why there was no difference in identifying VAPs according to profession, which could occur in other countries.

We acknowledge limitations in the study, including the inability to include all departments in Colombia, and we cannot guarantee that professionals answered the survey without assistance.

The significance of this study for daily clinical practice lies in providing evidence on ICU professionals’ ability to identify PVAs, a critical factor for optimizing mechanical ventilation and improving outcomes in critically ill patients. The findings underscore the need for specific training in this area, which could inform the development of targeted training programs and the implementation of complementary technologies, such as monitoring software, to enhance patient-ventilator synchrony and mitigate the negative impacts of PVAs in the ICU.

## Conclusions

A low percentage of ICU healthcare professionals in Colombia were able to correctly identify all PVAs. Factors significantly associated with accurate identification included receiving specific training in this area and working in mixed or neonatal ICUs.
